# Neoadjuvant Pembrolizumab Enables Successful Downstaging and Resection of Borderline Resectable MSI-H/dMMR Pancreatic Ductal Adenocarcinoma: A Case Report and Literature Review

**DOI:** 10.1007/s12029-025-01237-5

**Published:** 2025-05-08

**Authors:** Kevin Zhao, Vijayaragavan Muralidharan, Shaun Brown, Anthony Upton, Moammar Alshimirti, Prasad D. Cooray

**Affiliations:** 1 University of Melbourne School of Medicine, Melbourne, Australia; 2 Prometheus Research Collaborative, Department of Surgery, Austin Precinct, The University of Melbourne, Austin Health, Melbourne, Australia; 3 TissuPath, Mount Waverley, Australia; 4 Radiology Victoria, Melbourne, Australia; 5 Australian Clinical Labs, Clayton, Australia; 6 Department of Surgery, The University of Melbourne, Austin Health, Melbourne, Australia

**Keywords:** Neoadjuvant pembrolizumab, PDAC, MSI-H/dMMR

## Abstract

**Background:**

Pancreatic ductal adenocarcinoma (PDAC) is a highly aggressive malignancy with a poor prognosis. While immunotherapy has shown limited efficacy in most PDAC cases due to an immunosuppressive tumour microenvironment, tumours with microsatellite instability-high (MSI-H) or deficient mismatch repair (dMMR) status exhibit increased responsiveness to immune checkpoint inhibitors.

**Case Presentation:**

We report the case of a 45-year-old woman with Lynch syndrome who was diagnosed with MSI-H/dMMR PDAC during routine surveillance. Given the borderline resectable nature of her tumour and previous chemotherapy-related neurotoxicity, she was treated with neoadjuvant pembrolizumab instead of conventional chemotherapy. Following four cycles of pembrolizumab, imaging revealed a marked metabolic response, allowing for successful R0 pancreatoduodenectomy. Postoperative histology confirmed a significant reduction in tumour size, and immunohistochemical analysis demonstrated increased CD8 + T cell infiltration, supporting an enhanced anti-tumour immune response. The patient continues adjuvant pembrolizumab therapy without complications.

**Conclusion:**

This case highlights the potential role of neoadjuvant pembrolizumab in MSI-H/dMMR PDAC, demonstrating successful tumour downstaging and facilitating surgical resection. Our findings support further investigation into the integration of immunotherapy as a neoadjuvant strategy for select PDAC patients.

## Introduction

Accounting for over 500,000 cases and 400,000 deaths annually, pancreatic cancer is the 12 th most common cancer diagnosis, but the 6 th highest contributor to cancer mortality [1]. Amongst pancreatic cancers, pancreatic ductal adenocarcinoma (PDAC) is the most common, comprising over 90% of all pancreatic cancer cases [2]. PDAC is recognised as a disease of poor prognosis, and the 5-year overall survival rate is consistently reported to be approximately 10% or lower [3]. This poor prognosis of PDAC is partly attributable to late diagnosis, with the late onset of symptoms being a barrier against early detection [3].

Despite surgical resection being followed by a relatively high recurrence rate of 76.7% after 2 years [4], it is currently recognised as the only potentially curative modality for pancreatic cancer [5]. The treatment approach towards PDAC is dependent on disease stage and tumour resectability of the tumour, which is classified as resectable, borderline resectable, locally advanced, or advanced/metastatic. For patients with localised tumours, standard of care is upfront surgery followed by adjuvant chemotherapy FOLFIRINOX as a main standard, and gemcitabine-capecitabine being an alternative option. In this group, neoadjuvant chemotherapy/chemoradiotherapy may also be carried out [5]. In borderline resectable and locally advanced disease, neoadjuvant or induction chemotherapy/chemoradiotherapy can provide benefit, followed by resectability evaluations and surgical exploration where appropriate [5]. Agents used in these cases are FOLFIRINOX, gemcitabine plus nab-paclitaxel, or alternative gemcitabine-based combinations. For advanced and metastatic disease, chemotherapy or supportive/palliative approaches are taken depending on the patient’s functional status [5].

Complicating the treatment of PDAC is the representation of around 1% of cases by deficient mismatch repair (dMMR) or microsatellite instability-high (MSI-H) status [5]. Although this characterisation comprises only a small proportion of PDAC, tumours with MSI-H/dMMR status are associated with poorer responses to 5-fluorouracil (a component of FOLFIRINOX) and gemcitabine-based therapies [6], the standard chemotherapy agents used in the treatment of pancreatic cancers.

In recent years, immunotherapy has been explored as an option for cancer treatment. An immunotherapy agent that has been widely studied is pembrolizumab, an immune checkpoint inhibitor that targets PD-1 and is approved for site-agnostic use against MSI-H/dMMR cancers. When compared to 5-fluorouracil-based chemotherapies in the context of advanced MSI-H colorectal cancers, pembrolizumab has been associated with longer progression-free survival, a higher overall response rate, and a prolonged ongoing response [7, 8]. Pembrolizumab is also associated with prognostic benefits when used against non-colorectal MSI-H/dMMR cancers [9].

PDAC, being typically immunologically “cold” due to their immunoevasive properties, are often poorly responsive to immunotherapy [10]. However, pancreatic neoplasms with confirmed MSI-H/dMMR status have improved responsiveness to immunotherapy, enabling the exploration of immunotherapy as a therapeutic option in this group [11]. A cohort study investigating the efficacy of pembrolizumab suggests that it elicits a superior overall response rate compared to current first-line treatments FOLFIRINOX and gemcitabine plus nab-paclitaxel, and that its efficacy against MSI-H/d-MMR PDAC is comparable to that seen in MSI-H/d-MMR cholangiocarcinoma and gastric cancers [11]. Nevertheless, currently there is limited data suggesting the effectiveness of immunotherapy against MSI-H/d-MMR PDAC, and furthermore, very limited exploration of the use of pembrolizumab and immunotherapy in a neoadjuvant approach against borderline resectable MSI-H/dMMR PDAC [12] preceding surgical resection. Main neoadjuvant approaches currently remain chemotherapy, chemoradiotherapy, or a combination of the two [5]. The use of immunotherapy in a neoadjuvant approach is yet to be extensively explored.

This is a case report of a patient with Lynch syndrome and PDAC with confirmed MSI-H/d-MMR status, demonstrating the efficacy of immunotherapy pembrolizumab used to control and downsize the lesion for subsequent surgical resection in a neoadjuvant-adjuvant approach.

## Case Description

The patient was a 39-year-old, previously well professional artist when she developed gastric cancer in 2011. She had the symptoms of mild epigastric pain, increasing reflux symptoms, and nausea. She was found to have an ulcerating tumour in the gastric cardia region extending to the OG junction. Histology showed invasive undifferentiated carcinoma. Staging with CT and PET scans demonstrated the primary tumour measuring 6 cm and no evidence of metastasis. Laparoscopic washings were negative.

Patient was treated with preoperative epirubicin, cisplatin, and capecitabine (ECX regimen), which was the standard of care at the time. She had a total gastrectomy, splenectomy, and distal pancreatectomy in July 2011. Histology revealed poorly differentiated adenocarcinoma extending to perigastric fat, 1 of 26 lesser curve node positivity, and R0 margin negative resection. There was loss of MLH1 and PMS2, with preserved staining for MSH2 and MSH6, indicating microsatellite instability (MSI-H/d-MMR status). As there was poor response to preoperative treatment, she had postoperative FLOT chemotherapy (5 FU, leucovorin, oxaliplatin, docetaxel). Subsequently, upon germline testing, the patient was found to have Lynch syndrome.

In 2016, at the age of 45, she elected to have prophylactic TAH/BSO. The patient underwent regular colonoscopy screening. In 2023, she was found to have a high-grade dysplastic colonic lesion that was treated with EMR.

**Fig. 1:**
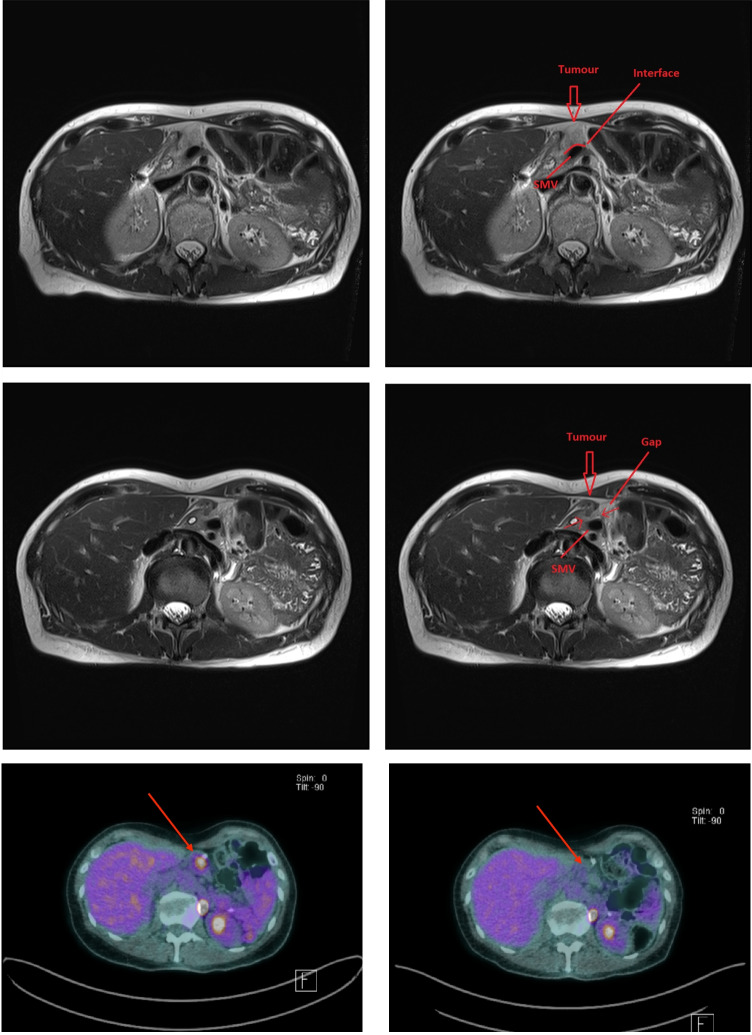
**a** Axial MRI images in July 2023 prior to treatment (top panels) and in October 2023 after 4 cycles of Pembrolizumab (bottom panels). Images demonstrate initial tumour contact with the SMV and a tissue plane after treatment. **b** Axial PET-CT image in July 2023 prior to treatment (left) and in October 2023 after 4 cycles of Pembrolizumab (right). A high-FDG avid lesion is seen in the pancreatic head region (arrow) with a complete metabolic response after treatment

In July 2023, on a surveillance CT scan, remnant pancreatic atrophy was noted. She reported unstable blood sugar levels over the preceding month, but no other symptoms nor weight loss. As a result, an MRI of the pancreas was done, which demonstrated a pancreatic head lesion measuring 24 mm with broad contact with the distal portal vein and SMV and, as a result, deemed borderline resectable (Fig. 1a). CA 19.9 was normal. An FDG-PET scan demonstrated activity in the same region with no metastatic lesions (Fig. 1b). EUS-guided biopsy of the lesion showed moderately differentiated adenocarcinoma, strongly positive for CK7 and CK20, and negative for CDX2. MMR protein immunohistochemistry showed loss of nuclear reactivity for MLH1 and PMS2, in keeping with Lynch syndrome and MSI-H/d-MMR status.

As it is the standard practice of our multidisciplinary team (MDT) to offer preoperative systemic therapy for patients with locally advanced/borderline resectable PDAC, we discussed neoadjuvant treatment options. FOLFIRINOX and gemcitabine/Nab-Paclitaxel chemotherapy were discussed as standard treatment options. Both regimens contain drugs that induce neurotoxicity (oxaliplatin and Nab-paclitaxel). As she was a painter by profession and had previous experience with FLOT chemotherapy-related neurotoxicity, she was averse to the proposed chemotherapy options. The efficacy of immunotherapy in MSI-H/d-MMR gastrointestinal cancers was discussed, and perioperative pembrolizumab was offered as an alternative strategy, but with limited data relevant to PDAC. The patient elected to proceed with pembrolizumab and received 4 cycles at a dose of 200 mg given intravenously every 3 weeks. We chose 4 cycles (12 weeks of treatment) as a balance point to allow adequate treatment to assess disease response and agility to allow a switch to chemotherapy if disease response were to be suboptimal.

In October 2023, following pembrolizumab treatment, repeat MRI imaging confirmed conversion from a borderline resectable to a resectable status (Fig. 1a). FDG PET-CT scan demonstrated markedly reduced activity in the region of the lesion (Fig. 1b).

In November 2023, the patient underwent pancreatoduodenectomy without complications.

### Operation

Staging laparoscopy was not performed due to very thin body habitus and multiple previous abdominal operations. A bilateral curved subcostal incision was followed by extensive division of postoperative adhesions and staging laparotomy and IOUS of the liver. The duodenum and pancreatic head were mobilised by radical Kocher’s manoeuvre, and dissection continued along the duodenum to expose the superior mesenteric vein, which was separated from the uncinate process. A retropancreatic tunnel was created. Only a small remnant of the body was present secondary to the distal pancreatectomy done as part of the previous gastrectomy. Porta hepatis dissection was performed as standard, and the common hepatic duct was transected. The duodenum was transected to the right of the SMV, and the specimen was removed. The jejunum was mobilised and pulled up to perform an end-to-side hepaticojejunostomy. The existing roux loop and gastrojejunostomy were left intact. Calculated blood loss was 95 ml. Patient was discharged 9 days after the operation.

### Pathological Findings

**Fig. 2:**
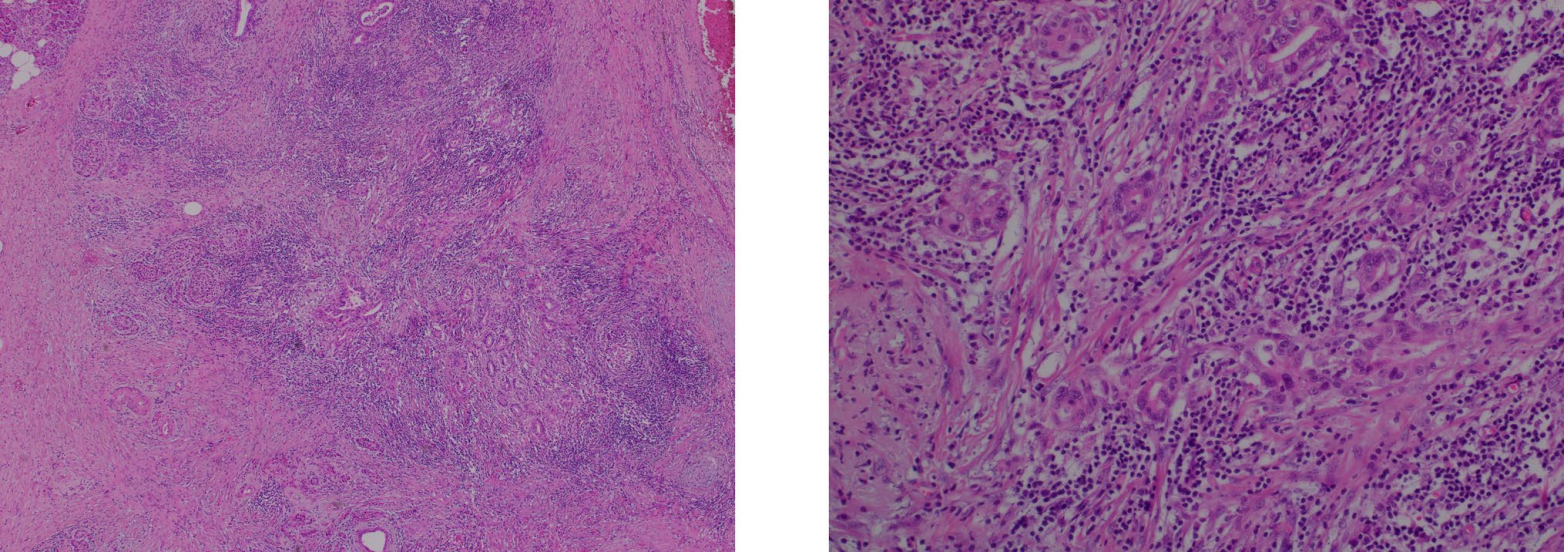

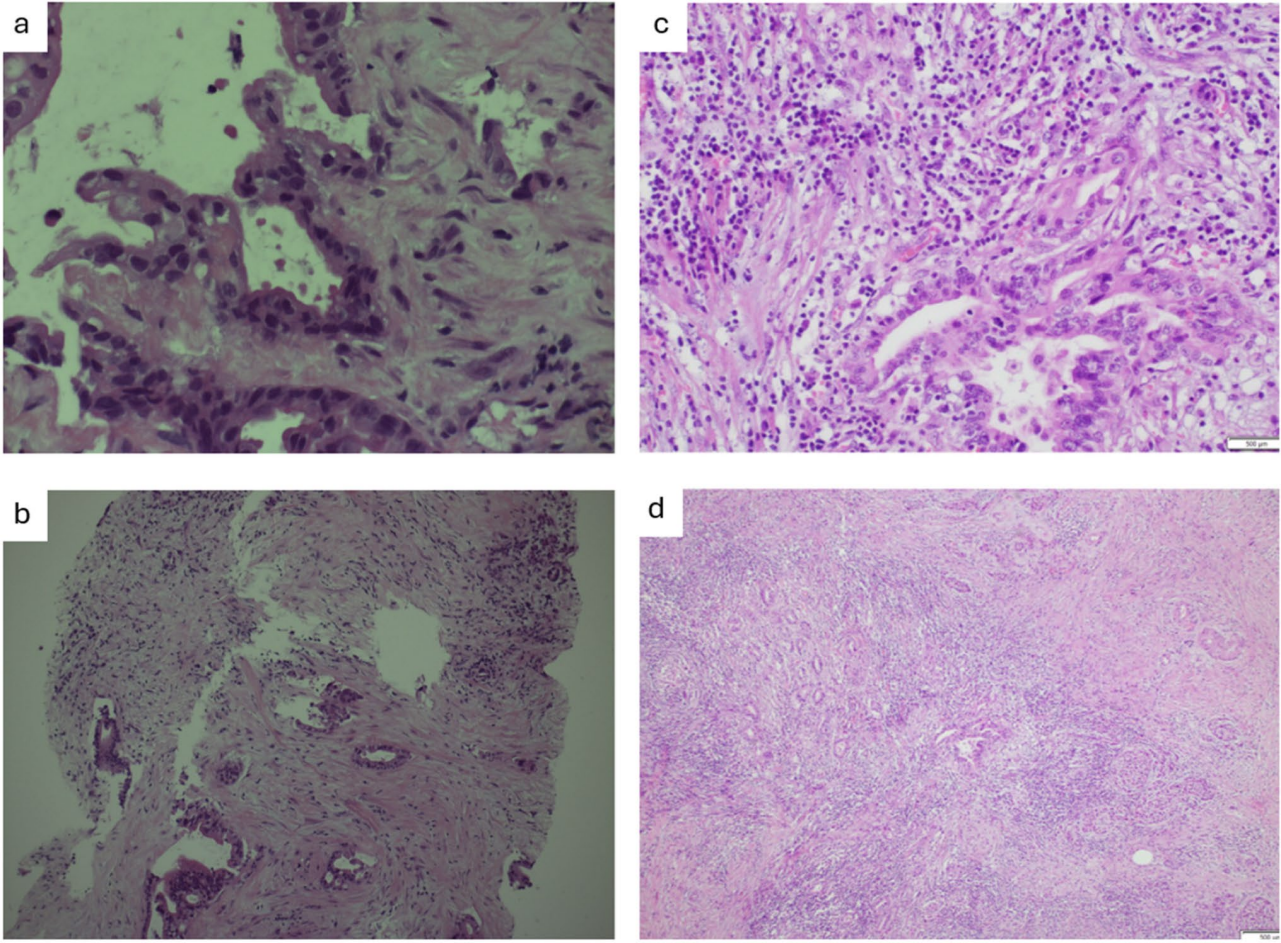
**a** Tissue H&E staining of the pancreatoduodenectomy specimen H&E staining 40x magnification (left panel). H&E staining 200x magnification (right panel). **b** Tissue H&E staining of the lesion. (**a**, **b**) H&E staining prior to treatment. (**c**, **d**) H&E staining postpembrolizumab, with increased lymphocytic infiltration compared to before treatment with pembrolizumab

**Fig. 3:**
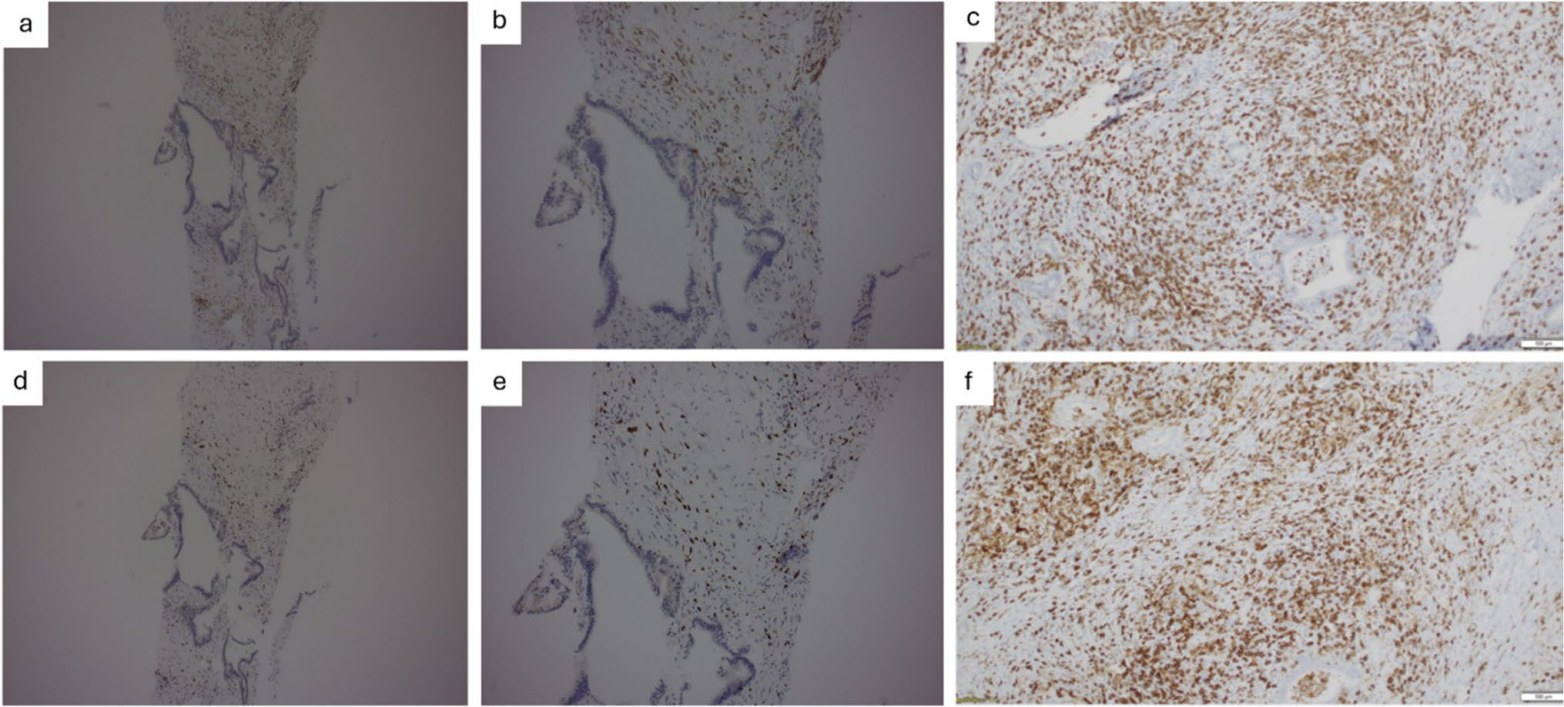
Immunohistochemistry analysis of the biopsied lesion pre- and post-treatment with pembrolizumab. (**a**) CD4+ T cell immunohistochemistry staining pre-treatment at 5x magnification. (**b**) CD4+ T cell immunohistochemistry pre-treatment at10x magnification. (**c**) CD4+ T cell immunohistochemistry staining after treatment. (**d**) CD8+ T cell immunohistochemistry pretreatment at 5x magnification. (**e**) CD8+ T cell immunohistochemistry pre-treatment at 10x magnification. (**f**) CD8+ T cell immunohistochemistry post-treatment. Increased intra-tumour CD8+ T cell infiltration is demonstrated

Histology demonstrated a 10-mm residual adenocarcinoma (ypT1 N0) with no nodal involvement and was margin-negative (R0) (Fig. 2a). Comparisons of tissue biopsies obtained before and after treatment with pembrolizumab on H&E staining (Fig. 2b), cell count (Fig. 3), and immunohistochemical analysis (Table 1) demonstrate increased CD8 + T cell infiltration and decreased CD4 +/8 + ratio.

**Table 1:** Intra-tumourinfiltrating CD4 + and CD8 + T cell counts before and after treatment with pembrolizumab. Post treatment, there is increased intra-tumour infiltration of CD8 + T cells, indicated by increased CD8 + cell count on microscopy and reversal of the CD4 +/CD8 + ratio

Tumour state	CD4 +/HPF	CD8 +/HPF	CD4 +/8 + ratio
Pre-Rx	140	100	1.4
Post-Rx	120	200	0.6

Following surgery, the patient commenced adjuvant pembrolizumab therapy (200 mg IV every 3 weeks), which she continues to date with no adverse effects. She remains relapse-free and well at a 20-month time interval from diagnosis.

## Discussion

We describe a patient who was found to have borderline resectable PDAC following surveillance CT, preceded by unstable blood glucose levels for 1 month on a background of Lynch syndrome in the absence of other symptoms. MRI demonstrated broad contact between the lesion and the portal vein and the superior mesenteric vein. Treatment, to good effect, followed a neoadjuvant approach with immune checkpoint inhibitor pembrolizumab. Existing literature describe pembrolizumab as being effective against locally advanced MSI-H/dMMR colorectal cancers [7, 8]. And in MSI-H/dMMR PDAC, it has been shown that pembrolizumab could successfully downstage MSI-H/dMMR PDAC become resectable by assessment [13].

Surgical resection remains the only potential curative approach towards pancreatic cancer [5]. The classification of pancreatic cancers into resectable, borderline resectable, and unresectable/locally advanced stages is based on the probability of achieving a margin-negative (R0) resection, including consideration for the degree of contact between the neoplasm and its surrounding veins and arteries. Due to the late onset of detectable symptoms, a majority of patients present with late stages of the disease that preclude them from direct surgical resection [2]. In the current patient, R0 resection was achieved following neoadjuvant immunotherapy treatment for four cycles, as described, which successfully downsized the lesion.

Neoadjuvant treatment is currently a recognised treatment option for borderline resectable PDAC. The rationale of this treatment is to improve the likelihood of an R0 resection and to aid early systemic control of the disease [12]. Agents that have thus far been extensively studied for use in conventional neoadjuvant treatments against PDAC are chemotherapies including FOLFIRINOX and gemcitabine plus nab-paclitaxel, with various other combination chemotherapies in early trials [12]. Immunotherapies, on the other hand, including agents that target PD-1/PD-L1-mediated immune evasion mechanisms, are often ineffective against most pancreatic cancers, which are frequently immunologically “cold”, infiltrated by suppressive immune cells and tumour microenvironments [10]. However, the small subset of pancreatic cancers that identify with MSI-H/dMMR status [5] is shown to be responsive to PD-(L)1 inhibition. MSI-H/dMMR tumours have a higher tumour mutational burden that generates an immunogenically “hot” environment, as evidenced by increasing CD4 + and CD8 + lymphocytic infiltration [14]. This formed the basis for selecting pembrolizumab as the neoadjuvant immunotherapy agent in the current patient, with the patient having known Lynch syndrome and immunohistochemical analysis of the tumour biopsy confirming deficiencies in MLH1 and PMS2.

A change in CD4 + and CD8 + dynamics after pembrolizumab treatment is demonstrated in the present case by a confirmed increase in CD8 + T cell infiltration and a reversal of the CD4/8 ratio post-treatment. While this area has not been extensively studied, emerging evidence nevertheless suggests that similar changes in CD4 + and CD8 + dynamics point to improved cytotoxic effector functions and are correlated with favourable outcomes and tumour response [15, 16].

In existing examples in which pembrolizumab was utilised against MSI-H/dMMR cancers, surgical resection and/or follow-up treatment with chemotherapy were not carried out at the times of reporting [8, 13]. To date, there are no examples within the literature that illustrate the complete course of a neoadjuvant immunotherapy followed by surgical resection against PDAC. This case report of our described patient serves as an example in which neoadjuvant pembrolizumab was utilised, followed by surgical resection and adjuvant immunotherapy. Following the initial course of neoadjuvant pembrolizumab, FDG-PET scan, MRI, CT, and histological analysis confirmed that the lesion was successfully downsized. In addition to imaging, the efficacy of immunotherapy pembrolizumab in the presented case was also demonstrated by the post-treatment increase in the infiltration of CD8 + T cells within the tumour and the decreased CD4 +/CD8 + ratio. The present case, therefore, demonstrates the efficacy of pembrolizumab as an immunotherapy used pre-operatively in the context of MSI-H/dMMR PDAC.

Despite multimodality treatments, borderline resectable PDAC has a poor prognosis. Barnes et al. reported a study of 185 real-life cases of borderline resectable PDAC patients [17]. Only 62% were able to complete the intended neoadjuvant therapy and surgery. The median survival was 20 months for the entire group and 31 months for the resected group. Maggino et al. reported 267 patients with borderline resectable PDAC, with only a 24% resection rate and a median survival of 12.8 months [18]. A more contemporary series of neoadjuvant FOLFIRINOX for borderline resectable PDAC demonstrated a median survival of 19.8 months [19]. Our patient, on the other hand, was able to be successfully downsized, the primary tumour was resected with a clear margin, and he is alive with no evidence of disease at 20 months from diagnosis.

The very favourable outcome of this patient to date reflects the close association between MSI-H/dMMR status of the tumour and its altered immunological characteristics, which corresponds to improved responsiveness to immunotherapy [14]. This can be leveraged clinically in neoadjuvant approaches against borderline-resectable and locally advanced MSI-H/dMMR PDAC, alternative to existing neoadjuvant modalities [5]. This concurs with the existing recognition that MSI testing can have significant clinical implications for informing treatment [5] and highlights the potential significance of MSI testing for all PDAC patients.

Overall, this case report demonstrates a clinical case in which the efficacy of immunotherapy pembrolizumab against MSI-H/dMMR tumours was leveraged in a neoadjuvant approach against immunogenically “hot” MSI-H/dMMR pancreatic ductal adenocarcinoma. Additional considerations that this case report highlights are the significance of MSI testing in clinical decision making, especially when conventional first-line chemotherapies are deemed less desirable.

## Data Availability

Sequence data that support the findings of this study are contained in the patient&apos;s electronic medical record (EMR).
